# Clinical Presentation and Genomic Analysis of HPV-Related Squamous Cell Carcinoma of the Larynx in Two Young Female Patients

**DOI:** 10.7759/cureus.48316

**Published:** 2023-11-05

**Authors:** Zahra Taboun, Peter Zeng, Jasna Deluce, Kevin Fung, John Barrett, Lama Elkadri, David Palma, Paul Stewart, Matthew J Cecchini, Anthony Nichols, Eric Winquist

**Affiliations:** 1 Medicine, Schulich School of Medicine and Dentistry, Western University, London, CAN; 2 Medical Oncology, London Health Sciences Center, London, CAN; 3 Otolaryngology - Head and Neck Surgery, Western University, London, CAN; 4 Otolaryngology - Head and Neck Surgery, London Health Sciences Center, London, CAN; 5 Radiation Oncology, Victoria Hospital, London Health Sciences Center, London, CAN; 6 Pathology and Laboratory Medicine, London Health Sciences Center, London, CAN

**Keywords:** exome sequencing, cancer genomics, laryngeal cancer, laryngeal carcinoma, hpv

## Abstract

Laryngeal cancer most frequently develops in males aged 60-70 years with a history of tobacco and/or alcohol use, while fewer cases occur in young patients in which tobacco and alcohol are often absent or less significant, highlighting the importance of other etiologies. We present cases of human papillomavirus (HPV)-associated laryngeal cancer in two previously healthy young women. A retrospective case review was carried out for both patients. DNA was extracted from the primary tumors and matched to normal tissue or blood, HPV genotype was determined by PCR and whole exome sequencing was carried out. Genomic results were pooled with laryngeal cancer patients from the cancer genome atlas (TCGA) dataset. The first patient was an 18-year-old female who underwent laryngectomy followed by adjuvant radiation. The second was a 24-year-old female who received chemoradiation. The first patient has remained disease-free for 16 years and the second for two years; both continue to be monitored. One tumor was positive for HPV45 and had mutations in FAT1 and FAT2; the other was positive for HPV31 and had mutations at NOTCH1, MAPK1, and HIST1H2AK. Both tumors had wild-type TP53 alleles. We bring attention to HPV as an etiology of laryngeal carcinoma in young patients, which may have implications for the treatment and prognosis of similar patients.

## Introduction

Laryngeal squamous cell carcinoma (LSCC) is one of the most common head and neck cancers, with 184,404 new cases and 88,840 deaths in 2020 worldwide [1]. LSCC most frequently occurs in males aged 60-70 years, with few cases (<10%) occurring in individuals younger than age 40 [2]. In the general demographic of patients, significant risk factors for LSCC include tobacco and alcohol use, however, exposure to these risk factors is often absent or minimal in individuals who present at a younger age [3]. Furthermore, carcinogenic exposures such as smoking tend to be dose-related and have a long latency period prior to the development of cancer, which is why smoking-related cancers develop later in life. In individuals that have no history of alcohol or tobacco use, other risk factors for LSCC include environmental exposures, gastroesophageal reflux, viral infections (human papillomavirus (HPV), human immunodeficiency virus (HIV), herpes simplex virus (HSV)), dietary factors, radiation, and individual predisposition [4].

HPV has been associated with an increased incidence of all head and neck cancers and is more frequently associated with oropharyngeal squamous cell cancers (OPSCC) [5]. Patients with HPV-associated OPSCCs experience markedly improved survival after treatment compared with HPV-negative patients [6-8], to the extent that it was necessary to develop a separate staging system for this distinct disease [9,10]. We present two cases and the tumor genetic profiles of young women (aged 18 and 24) diagnosed with LSCC caused by non-HPV 16 types. We further compare these genetic profiles with LSCC cases from the cancer genome atlas (TCGA).

## Case presentation

Case 1

An 18-year-old female presented with a six-year history of hoarseness and a one-year history of sore throat, difficulty swallowing, and weight loss. She had no other comorbid medical conditions, was a non-drinker, and non-smoker, and had no significant exposure to second-hand smoke. She had no history of acid reflux, no previous HPV diagnosis, no history of high-risk sexual behavior, no previous pap tests were completed, and she had not received a previous HPV vaccine. Although her grandmother had a history of thyroid cancer, there were no other instances of head and neck cancers in the family.

**Figure 1:**
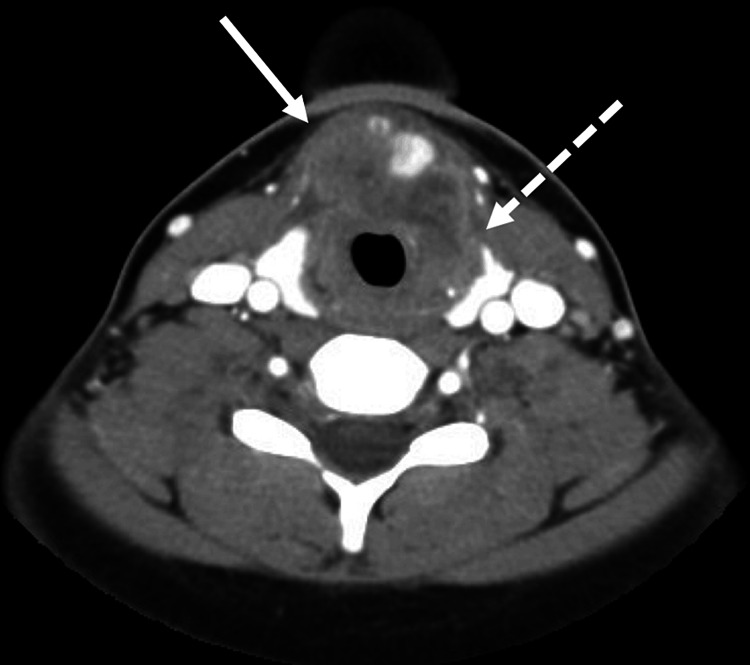
Axial view CT scan of the neck demonstrated the tumor invading the anterior larynx (solid arrow) and thyroid gland (dashed arrow).

**Figure 2:**
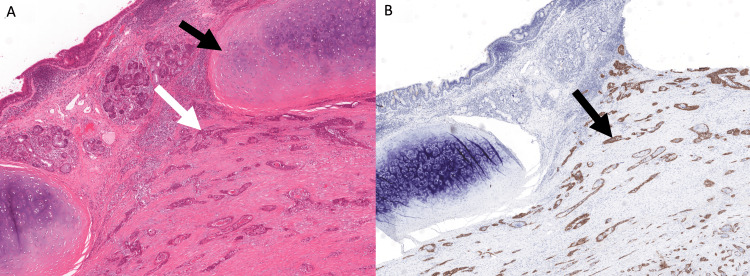
Pathology results for case 1. (A) Squamous cell carcinoma involving the submucosal tissues (white arrow) and diffusely infiltrating under the cartilage (black arrow). (B) Tumor cells (black arrow) diffusely and strongly positive for p16 by immunohistochemistry.

Computed tomographic (CT) scan of the head and neck showed a 5.0 cm mass arising below the hyoid bone and extending to the isthmus of the thyroid and the anterior vocal cords with destruction of the thyroid cartilage (Figure 1). There was no evidence of adenopathy to suggest metastasis to lymph nodes. A laryngoscopy was performed and there was no evidence of an obvious mucosal lesion; however, the right vocal cord was paralyzed suggesting probably invasion of the nerve. An open neck biopsy of the larynx was done, and the pathology showed p16-positive keratinizing moderately differentiated invasive squamous cell carcinoma (Figures 2A, 2B).

The patient underwent a total laryngectomy, bilateral neck dissection, and total thyroidectomy. Pathology described a 1.5 x 1.5 x 1.0 cm invasive squamous cell carcinoma of the right vocal cord that involved the soft tissue anterior to the thyroid and the thyroid capsule, the submucosal layer of the right and left subglottic and supraglottic regions, and suprahyoid soft tissues. There were three positive lymph nodes in the right neck, with the largest measuring 2.0 cm with no extranodal extension. The final pathological stage was IVA (pT4N2bM0) according to the AJCC 8th edition (AJCCV8) staging system.

The patient received postoperative chemoradiation. Radiation therapy consisted of 6600 cGy in 31 fractions using intensity-modulated radiation therapy with three cycles of concurrent cisplatin chemotherapy (100mg/m^2^ IV) given every three weeks with the third cycle omitted due to intolerable nausea and vomiting. Overall, the patient recovered well from surgery without complications and tolerated radiation therapy with only a modest weight loss. The patient has been on surveillance without recurrence for the past 16 years.

Molecular testing including genomic sequencing and HPV subtyping (see Appendix for details on methodology). Samples obtained from this patient were positive for HPV45 and confirmed by Sanger sequencing. A total of 312 mutations were identified in the tumor’s whole exome sequences when compared to the patient’s control DNA, including mutations in the FAT1 and FAT2 genes, and the tumor was wild type for TP53.

Case 2

A 24-year-old female presented with a one-year history of persistent and progressive hoarseness. She had a history of anxiety, depression, tonsillectomy in childhood, and tubal ligation at the age of 21. She had no previous HPV diagnosis, no history of high-risk sexual behavior, no previous pap tests were completed, and she had not received a previous HPV vaccine. There was no known family history of cancer, although she had a 10-pack-year smoking history.

**Figure 3:**
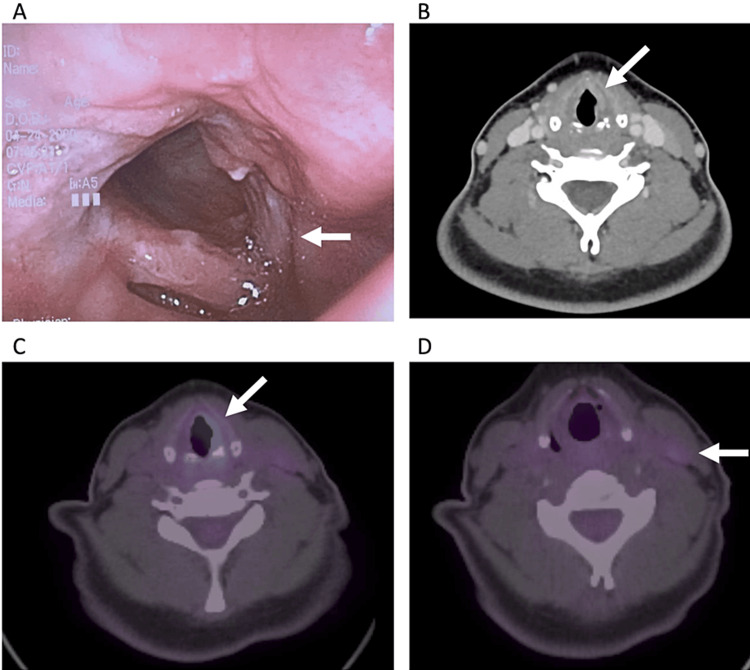
Tumor images obtained for case 2. (A) Naopharyngoscopy demonstrated the left vocal cord lesion (blocked arrow) extending to the anterior commissure. (B) CT imaging of the primary tumor (blocked arrow) with anterior commissure involvement (blocked arrow). PET CT images demonstrating the mildly PET avid primary tumor (blocked arrow) (C) and left neck node (blocked arrow) (D).

The patient underwent nasopharyngoscopy which revealed a lesion with an epicenter of the left posterior half of the true vocal fold extending to the anterior commissure (Figure 3A). No subglottic extension was reported, and the cord was mobile. CT of the head and neck showed soft tissue fullness involving the left true cord estimated to measure 1.5 cm x 0.55 cm x 1.1 cm (Figure 3B). This extended to the lateral ventricle superiorly, into the subglottic larynx inferiorly, and to the commissure. Furthermore, there was moderate fullness at the tongue base and enlarged lymph nodes bilaterally. The positron emission tomography (PET) CT scan demonstrated a mildly acid primary tumor (Figure 3C) and lymph nodes (Figure 3D).

**Figure 4:**
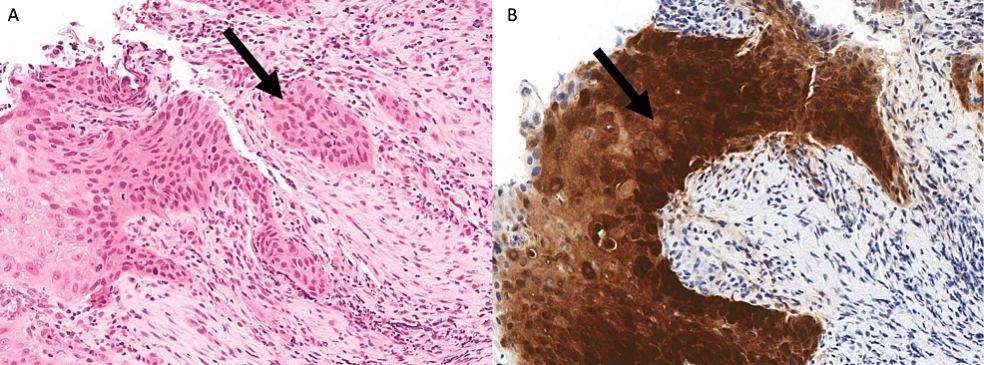
Pathology slides for case 2. (A) Squamous cell carcinoma with underlying stromal invasion (black arrow). (B) Tumor cells diffusely and strongly positive for p16 by immunohistochemistry (black arrow).

A panendoscopy and biopsy of the lesion on the left vocal revealed p16-positive squamous cell cancer (Figures 4A, 4B). PET whole-body imaging showed moderate F-fluorodeoxyglucose (FDG) uptake in the left true vocal cord and mild uptake in a left level three node. Fine needle aspiration biopsy of the left level III node reported reactive changes without evidence of malignancy. The patient was deemed to have stage III (cT3N0M0, AJCCV8) squamous cell carcinoma of the left vocal cord. Radical radiation therapy with concurrent chemotherapy was recommended. She received three cycles of cisplatin (100 mg/m^2^ IV) given every three weeks concurrent with external beam radiotherapy 70 Gy in 35 fractions. The patient has experienced no recurrences in the three years after treatment and continues to do well.

Molecular testing including genomic sequencing and HPV subtyping was done (details on Methods can be found in appendices). Samples obtained from this patient were positive for HPV31 by PCR and confirmed by Sanger sequencing and alignment to HPV31 genome sequences in Genbank. A total of 95 mutations were identified in the tumor when compared to the patient’s control DNA, including mutations in the following genes: NOTCH1, MAPK1 (ERK2), and HIST1H2AK, but was wild-type for TP53.

Statistical analyses

**Figure 5:**
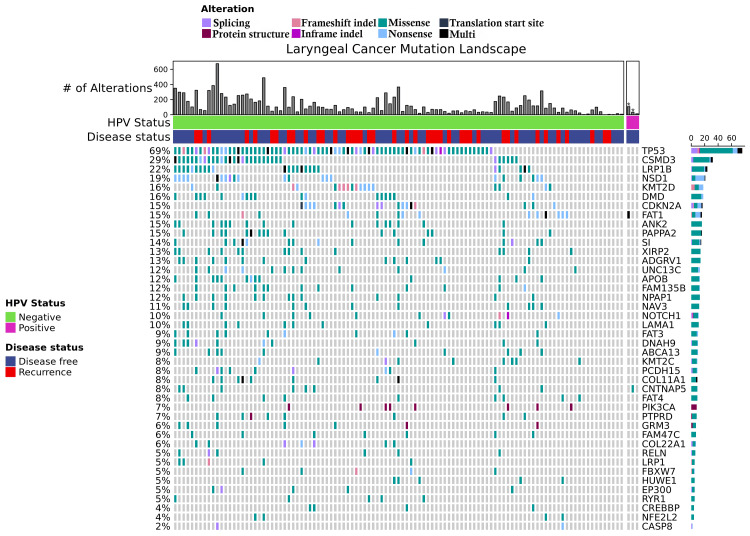
Pooled genomic analysis of HPV-positive versus negative LSCC with the TCGA. Asterisks indicate patient cases presented in this case report, with patient 1 on the left and patient 2 on the right. LSCC - Laryngeal squamous cell carcinoma, TCGA - the cancer genome atlas

We have previously examined the LSCC cohort of the TCGA [11]. After reviewing the pathology reports and availability of HPV status using RNA sequencing there are 108 LSCC patients, which were pooled with our two cases [11]. We compared the demographic factors of the three HPV-positive cases with the 107 HPV-negative cases and found no significant differences between the two groups (Table 1). We then compared the genomic landscape of the HPV-positive and negative laryngeal cancer cases, and we found that mutations present in HPV-negative LSCC tumors tended to be absent in the HPV-positive tumors (Figure 5).

**Table 1:** Comparison of demographic factors in HPV-positive and negative disease. FDR correction represents p-values obtained after correction with Benjamini-Hochberg method. Represented p-values were obtained using Fisher's Exact Test. NA: non-applicable.

		HPV-negative (n = 107)	HPV-positive (n = 3)	P-value	FDR
Age (median, sd)	-	62 (9.3)	24 (24.4)	0.065	0.217
Gender	Male	89	1	0.085	0.217
	Female	18	2		
Smoker	Yes	6	1	0.25	0.437
	No	98	2		
	NA	3	0		
Alcohol abuse	Yes	15	0	0.093	0.217
	No	24	2		
	NA	68	1		
T stage	1	3	0	0.785	0.916
	2	16	0		
	3	33	2		
	4	51	1		
	NA	4	0		
N stage	0	54	1	0.455	0.637
	1	16	1		
	2	25	1		
	3	2	0		
	NA	1	0		
M stage	0	100	3	1	1
	1	2	0		
	NA	5	0		

## Discussion

HPV is recognized as an important etiological factor in OPSCC, and HPV-associated OPSCC has a more favorable response to chemoradiation that is now recognized with a separate staging system for these cancers in the 8th edition of the American Joint Committee on Cancer (AJCC) staging system [7,10,11]. For patients with HPV-associated loco-regionally advanced OPSCC treated with chemoradiation therapy, five-year overall survival rates exceed 80%, while five-year survival for those with HPV-negative tumors receiving similar treatment is about 40% [7]. In view of this, treatment de-escalation intended to reduce long-term residual treatment effects while maintaining a high cure rate is an area of intensive research in HPV-associated OPSCC.

Although HPV status is widely recognized as an etiology and prognostic factor for OPSCC, the same is not clearly recognized for LSCC. A meta-analysis conducted by Torrente et al. showed that approximately 25% of laryngeal cancers may be HPV-related [12]. It seems plausible that laryngeal cancer in young individuals -- especially nonsmokers -- has a different biology and prognosis, and this may be related to HPV. Prior smaller studies showed mixed results, some demonstrating improved outcomes for patients with HPV-positive LSCC and others showing no difference [13]; in meta-analyses of the published literature, Sahovaler et al. reported improved overall survival in HPV-associated laryngeal cancer [14]. Further work is warranted to determine whether HPV status should be incorporated into the staging of laryngeal carcinomas.

It is well recognized that different HPV strains pose different risks for developing malignancy. Although HPV16 is the most common subtype of HPV associated with head and neck cancers, the cases presented in this case report highlight the role of other HPV subtypes in laryngeal carcinoma. Ndiaye et al. conducted a meta-analysis on HPV-associated laryngeal carcinomas and found that HPV16 was the most common subtype associated with these carcinomas worldwide followed by HPV18 and HPV6; however, stratification by age was not reported [15]. Interestingly, there was geographic variation in the prevalence of various subtypes; although HPV16 was the most common subtype in all geographic regions analyzed, the second and third most common subtypes varied by region [15]. Notably, there were no North American cases included in the meta-analysis that were associated with either of the subtypes presented in this case report (HPV45 and HPV31), highlighting the rarity of these cases, but also, emphasizing the importance of considering subtypes beyond those classically associated with laryngeal carcinoma. HPV31 is a member of the alpha-papillomaviruses species 9 group for which HPV16 is the representative “type” member; this means HPV31 is more similar to HPV16 and its other high-risk members of species 9 group (including HPV 33, 35, 52, 58, and 67) than it is to other HPVs including HPV18 or the low-risk HPV 6 and 11 [16]. In contrast, HPV45 is also an alpha-papillomavirus but a member of species 7 for which HPV18 is the “type” representative and includes HPV 39, 58, 68, and 70 [16].

In addition to tumor HPV status being discussed as a prognostic factor in head and neck cancers, the role of the specific HPV subtype on prognosis in HNC has also been explored in the literature to only a limited degree, likely because HPV types may not be routinely collected in pathology testing. There currently is no consensus on the impact of subtypes on prognosis. Bratman et al. found that patients with tumors associated with HPV16 had a better prognosis than those infected with other subtypes, with patients with HPV16-associated tumors having a three-year survival rate of 88% vs. 49% in those associated with other HPV subtypes (p=0.003) [17]. Conversely, Ziai et al. found no difference in five-year overall survival or progression-free survival in patients with HPV16-associated tumors compared to those with other subtypes (62.1% vs. 88.9%, p=0.273; 49.0% vs. 88.9%, p=0.081, respectively); however, results did approach significance for five-year progression-free survival [8]. Given the wide variety of HPV subtypes and the conflicting results in the literature, serotyping HPV-associated laryngeal carcinomas is important for advancing knowledge on whether HPV subtypes play a role in prognosis.

While the mutational burden of HPV-negative and HPV-positive head and neck squamous cell carcinomas (HNSCC) are similar, the genomics of the two groups differ in mutational makeup [18], which is consistent with the findings presented here. HPV-positive HNSCC tumors have been shown to harbor unique mutations in DDX3X and FGFR2/3 and enrichment of aberrations in PIK3CA, KRAS, MLL2/3, and NOTCH1 [18]. In this analysis of the TCGA cohort as well as other studies in the literature, the most common mutation present in HPV-negative LSCC tumors was TP53 (an important tumor suppressor), however, in general, HPV-positive tumors tend to have wildtype TP53 alleles [18-20]. Studies addressing the role of HPV on the complexity of genomic alterations and other genomic mechanisms (e.g., methylation) on prognosis in LSCC will help determine the role of HPV on prognosis.

In addition to ingested carcinogens, the risk of developing laryngeal carcinoma is higher in males and with advancing age. HPV-related head and neck cancers tend to be more common in men than in women; however, it is thought that gender differences in lifestyle and sexual behavior alone are not sufficient to explain the difference in the prevalence of HPV-related head and neck cancers between sexes [21]. It is hypothesized that hormones likely play a role in protecting females from head and neck cancers, as both estrogen- and progesterone-related factors are inversely related to the risk of developing head and neck squamous cell carcinoma [22]. Interestingly, our results presented in this study did not show a significant difference between sexes; this is likely due to the low sample size of the HPV-positive group (n=3) reducing the statistical power of the study. A larger sample size may help elucidate patterns more clearly.

As with other cancers, the risk increases with age, likely due to the accumulation of mutations over time. However, in HPV-associated cervical cancer, it was found that age alone was not an important factor, but rather, the duration of HPV infection and HPV subtype influenced the risk of progressing to cancer [23]. These data emphasize the difference in etiology between HPV and smoking/alcohol as causes of cancer and may offer an explanation as to why HPV-associated laryngeal cancer may develop more frequently and with less common HPV subtypes in younger individuals than smoking/alcohol-associated laryngeal cancer. Furthermore, the risk of developing laryngeal carcinoma at a young age as well as the protection that vaccines offer against high-risk HPV subtypes may impact vaccination schedules.

## Conclusions

This case report highlights the importance of considering HPV as an etiological factor in laryngeal carcinoma in young patients and emphasizes the importance of considering relatively rare HPV subtypes. The HPV subtypes implicated in both of our cases are included in newer HPV vaccinations, and therefore vaccination at an early age is likely beneficial in preventing HPV-associated laryngeal carcinomas. It is important to consider routinely testing all laryngeal carcinomas for HPV status and subtype, particularly in patients who are younger or do not have a history of carcinogen exposure, to further knowledge about the biology of this disease. Further work assessing tumor HPV status and subtype as a prognostic factor and potential factor in staging is warranted.

## Appendices

Pathology review, p16 immunohistochemistry, and DNA extraction

H&E slides were retrieved and reviewed by a head and neck pathologist (MC).  P16 immunohistochemistry was performed as we have previously described with a positive result being strong staining in >70% of tumor cells [24]. A five-micron thick slice (tissue curl) was taken from the formalin-fixed paraffin-embedded (FFPE) from patient 1’s tumor and non-diseased lymph node tissue, and from the tumor of patient 2. The FFPE tissue curls were deparaffinized using a deparaffinization solution (Qiagen) and genomic DNA was extracted using a QIAamp DNA FFPE Tissue Kit (Qiagen).  Genomic DNA was extracted from the leukocytes of patient 2 using a QIAamp DNA Blood Mini Kit (Qiagen).

HPV genotyping via polymerase chain reaction (PCR)

Patient genomic DNA was screened for the presence of high-risk HPV sequences using a range of high-risk HPV-type specific primers (HPV-16, HPV-18, HPV-31, HPV-33, HPV-35, HPV-39, HPV-45, HPV-51, HPV-52, HPV-55, HPV-56, HPV-58, HPV-67, HPV-68b, HPV-69, HPV-74).  Amplification of PCR products using HPV-31 and HPV-45 primers were gel-purified and Sanger sequenced at the DNA Sequencing Facility at Robarts Research Institute (London, ON). Forward/Reverse primer sequences for HPV31 were AACAACATTAGAAAAATTGACA/AGTACGAGGTCTTCTCCAACATGC. Forward/Reverse primer sequences for HPV45 were TCAAACTCTGTATATGGAGAG/GCCTGGTCACAACATGTATT. Sequences were aligned to HPV sequences in the NCBI Genbank database to confirm their identity.

Whole exome sequencing

Tumor samples and matched normal blood tissue sequenced were subject to whole exome sequencing at BGI (Hong Kong) using the DNBseq platform. Raw FASTQ files were aligned to the GRCh38 human reference genome, including available ALT and decoy alleles using BWA-mem (v0.7.17). Duplicate reads were marked using The Genome Analysis Toolkit (GATK v4.17.0). GATK was used for base quality recalibration, with regions limited to those targeted by the applicable exome capture kit. Coverage was estimated using BamQC. Somatic SNVs were identified using MuTect2 (v4.17.0) with according to GATK best practices using default parameters. To account for FFPE artifacts, additional sequence quality-based metrics were employed for filtering: identified somatic SNVs with a mutant allele fraction (MAF) ≥10%, covered by ≥15 reads in the normal and the tumor, and <2 reads supporting the mutation in the normal, an average base quality ≥20, an average mapping quality ≥56, and an average position on the read as a fraction ≥0.4 or a tumor MAF ≥20% were kept. Mutations were annotated using SnpEFF. HPV genotype was determined using HPViewer.
